# Systemic Evidence for Mitochondrial Dysfunction in Age-Related Macular Degeneration as Revealed by mtDNA Copy Number Measurements in Peripheral Blood

**DOI:** 10.3390/ijms242216406

**Published:** 2023-11-16

**Authors:** Adriana Koller, Claudia Lamina, Caroline Brandl, Martina E. Zimmermann, Klaus J. Stark, Hansi Weissensteiner, Reinhard Würzner, Iris M. Heid, Florian Kronenberg

**Affiliations:** 1 Institute of Genetic Epidemiology, Medical University of Innsbruck, 6020 Innsbruck, Austria; adriana.koller@i-med.ac.at (A.K.); claudia.lamina@i-med.ac.at (C.L.); hansi.weissensteiner@i-med.ac.at (H.W.); 2 Department of Genetic Epidemiology, University of Regensburg, 93053 Regensburg, Germany; caroline.brandl@ukr.de (C.B.); martina.zimmermann@ukr.de (M.E.Z.); klaus.stark@klinik.uni-regensburg.de (K.J.S.); iris.heid@klinik.uni-regensburg.de (I.M.H.); 3 Department of Ophthalmology, University Hospital Regensburg, 93053 Regensburg, Germany; 4 Institute of Hygiene and Medical Microbiology, Medical University of Innsbruck, 6020 Innsbruck, Austria; reinhard.wuerzner@i-med.ac.at

**Keywords:** mitochondrial DNA copy number, mtDNA abundance, age-related macular degeneration, aging, geographic atrophy

## Abstract

Mitochondrial dysfunction is a common occurrence in the aging process and is observed in diseases such as age-related macular degeneration (AMD). Increased levels of reactive oxygen species lead to damaged mitochondrial DNA (mtDNA), resulting in dysfunctional mitochondria, and, consequently, mtDNA causes further harm in the retinal tissue. However, it is unclear whether the effects are locally restricted to the high-energy-demanding retinal pigment epithelium or are also systematically present. Therefore, we measured mtDNA copy number (mtDNA-CN) in peripheral blood using a qPCR approach with plasmid normalization in elderly participants with and without AMD from the AugUR study (n = 2262). We found significantly lower mtDNA-CN in the blood of participants with early (n = 453) and late (n = 170) AMD compared to AMD-free participants (n = 1630). In regression analyses, we found lower mtDNA-CN to be associated with late AMD when compared with AMD-free participants. Each reduction of mtDNA-CN by one standard deviation increased the risk for late AMD by 24%. This association was most pronounced in geographic atrophy (OR = 1.76, 95% CI 1.19–2.60, *p* = 0.004), which has limited treatment options. These findings provide new insights into the relationship between mtDNA-CN in blood and AMD, suggesting that it may serve as a more accessible biomarker than mtDNA-CN in the retina.

## 1. Introduction

Age-related macular degeneration (AMD) causes central vision impairment in millions of elderly people worldwide, and the pathogenesis of this multifactorial disease is not well understood [1,2,3]. In epidemiological studies, a diagnosis of AMD is typically confirmed via gradable color fundus images. There is neither a cure nor an effective treatment for all types of AMD. The clinical endpoint of the disease is defined as irreversible vision impairment due to two subtypes of advanced disease: geographic atrophy or macular neovascularization. Macular neovascularization is characterized by the formation of new fragile vessels, triggered by the release of vascular endothelial growth factor (VEGF), among other factors, and vascular leakage causing damage of the surrounding tissue (wet AMD) [3,4,5]. Anti-VEGF treatment can slow down disease progression in patients with wet AMD. Geographic atrophy (also called dry AMD) appears with a degeneration of photoreceptors and retinal tissue. Currently, various preclinical studies based on complement inhibitors and stem cell transplantation are ongoing, but especially for geographic atrophy, there is still a strong need for therapeutic options [6,7,8,9,10,11]. Several questions regarding the etiology of AMD remain to be answered. Age, smoking and nutrition are known risk factors for developing AMD [12,13], and previously, a genome-wide association study identified over 50 variants to be associated with this disease [14]. Oxidative stress, inflammation and especially mitochondrial dysfunction are increased not only with progressing age but especially in AMD [15,16]. Different mitochondrial DNA (mtDNA) haplogroups as well as certain variants in the mtDNA are associated with AMD and have different impacts on developing this disease. Haplogroup H seems to have a protective effect against AMD, while others (for example haplogroups J and T) increase the risk for AMD drastically [17,18,19,20,21].

Human mtDNA is a small circular genome (16,569 base pairs) with 2–10 copies per mitochondrion [22,23]. The amount of mtDNA per cell can vary tremendously, depending on the oxidative stress, the energy demand of a cell and tissue and the presence of disease. Mitochondrial DNA is more affected by oxidative damage than nuclear DNA (nDNA), due to the limited availability of mtDNA repair mechanisms, among other things [24,25]. Therefore, the mtDNA copy number (mtDNA-CN) was investigated as a potential biomarker for mitochondrial dysfunction [26]. The mtDNA content is altered by imbalances in the energy metabolism [26,27], and changes in the mtDNA-CN are associated with several diseases [28,29,30,31,32]. The mechanisms of copy number regulation are mainly unknown, due to the multifaceted nature of mitochondria [33]. This is due to its tissue-specific function, its highly dynamic networks changing morphology in response to metabolic alterations, and its involvement in a plethora of processes, such as signaling processes, the mtDNA–nDNA crosstalk or its role in inflammation pathways [34,35]. 

The concept that mitochondrial dysfunction is involved in AMD, especially in the retina, has gained wide acceptance [15]. The available literature in the context of mtDNA-CN and AMD is based on cell culture experiments focusing on the local effects of mitochondria and mtDNA in retinal tissue [36,37,38]. However, mtDNA-CN in peripheral blood has not yet been investigated for AMD in epidemiological studies. Therefore, the objective of this study was to investigate the association of mtDNA-CN in the blood of individuals with and without AMD from a large population-based study of elderly individuals. 

## 2. Results

**Figure 1:**
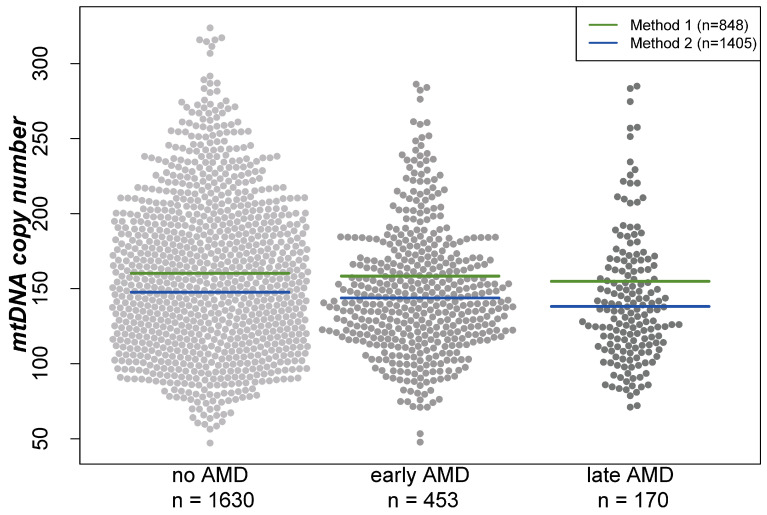
Distribution of mitochondrial DNA copy number in different groups visualized in a beeswarm plot. Lines indicate the mean in the respective groups depending on the DNA extraction method. “Method 1” represents the DNA extraction of samples using the Puregene reagents, while “Method 2” is based on a similar manual salting-out protocol. For both extraction methods, the lowest mtDNA-CN was found in individuals with late AMD and the highest in AMD-free participants.

Analyses were performed based on cross-sectional data from 1630 participants without AMD, 453 with early AMD and 170 with late AMD (geographic atrophy and/or neovascular complications) based on the Three Continent Classification [39]. Baseline characteristics of the participants of the AugUR study are provided in Table 1. When comparing individuals of these three groups, mtDNA-CN was the lowest in individuals with late AMD and the highest in participants without AMD (mean mtDNA-CN = 152.3, 147.8, or 142.0, respectively; Kruskal–Wallis test *p* = 0.002 for any difference in the three groups). Visualization of the mtDNA-CN distribution between the three groups showed that the differences in the means are similar for both extraction methods (beeswarm plot, Figure 1). Furthermore, women had a higher mean mtDNA-CN compared with men (156.08 versus 144.55, respectively; Wilcoxon rank-sum test *p* < 0.001). 

### 2.1. Mitochondrial DNA Copy Number and AMD

**Figure 2:**
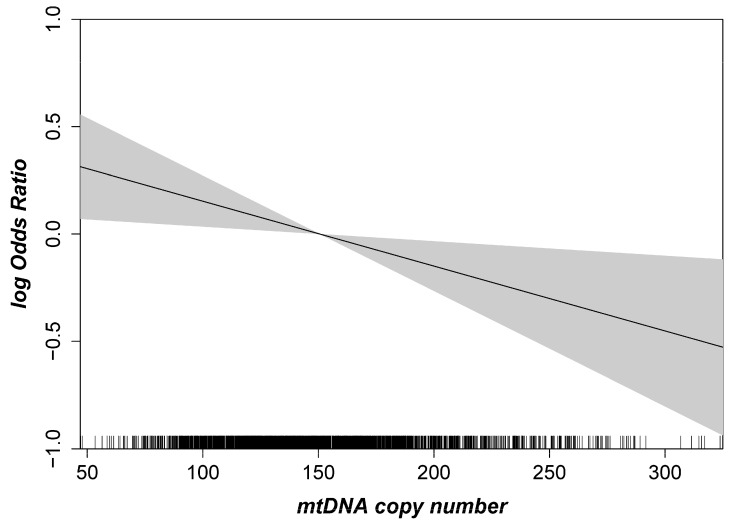
Age- and sex-adjusted logistic regression spline with given 95% confidence interval highlighting the association between mtDNA copy number and AMD. The mtDNA copy number is shown on the x-axis, and the logarithmic Odds Ratio (OR) for AMD is given on the y-axis (n = 2253).

First, we were interested in the association of mtDNA-CN with early or late AMD overall and stratified by sex. The spline based on a logistic regression model showed a linear inverse association between mtDNA-CN and AMD (Figure 2). As a consequence, mtDNA-CN was included as a continuous variable in the subsequent multinomial mixed-effect models with random effects to account for the extraction method. For the main model (adjusted for age, sex, smoking and blood cell counts), we found a significant association of mtDNA CN with late AMD, but not with early AMD: We observed a 24% higher odds for late AMD per one standard deviation (SD) lower mtDNA-CN (OR = 1.24, 95% CI 1.04–1.47, *p* = 0.02; one SD in mtDNA-CN = 41.5 copies). As shown previously, mtDNA-CN measurements in peripheral blood might be influenced by the blood cell counts [40]. Therefore, we applied different adjustment models, including leukocyte and thrombocyte counts. In our study, the estimates and *p*-values remained stable when adjusting the mtDNA-CN from peripheral blood for blood cell counts and upon further adjustment for cardiometabolic diseases and parameters (Table 2, Model 3–6). Sensitivity analysis using the formula described by Hurtado-Roca and colleagues [40] showed the same results for late AMD (OR = 1.22, 95% CI 1.02–1.44, *p* = 0.03; adjusted for age, sex and smoking). Thus, we found a significant association for late AMD that was robust upon various adjustment models.

We observed that the association between mtDNA-CN and late AMD vs. no AMD was stronger in men than in women (OR = 1.45, 95% CI 1.11–1.90 vs. 1.07, 95% CI 0.84–1.36 in men and women, respectively; Table 2, model 3). This was robust upon different adjustments in the model. However, when including an interaction term for mtDNA-CN and sex for late AMD, we found no statistically significant interaction (*p* = 0.09, adjusted for age, smoking, leukocyte and thrombocyte counts). The mtDNA-CN distribution between men and women is shown in a beeswarm plot in Figure S1, showing a similar trend between men and women when comparing no vs. early vs. late AMD. 

### 2.2. Effects Differ by AMD Subtypes 

**Figure 3:**
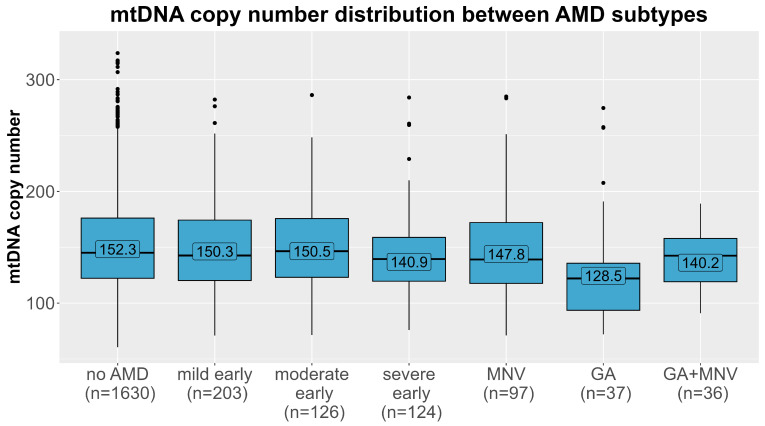
Distribution of mtDNA copy number (y-axis) in each age-related macular degeneration subtype (x-axis). Means of each AMD group (no AMD, mild/moderate/severe early AMD, macular neovascular AMD (MNV), geographic atrophy (GA) and the combined subtype) are labelled.

The mtDNA-CN levels differed significantly between the different AMD subtypes (Figure 3, Kruskal–Wallis test *p* = 0.0015). We further tested whether this association was present in all subtypes of AMD in multinomial regression analyses in the main model (adjusted for age, sex, smoking, and leukocyte and thrombocyte counts, model 3). As before, the estimates were robust upon different adjustments in the model (Table S1). A strong significant association was observed for geographic atrophy (n = 37), with almost a doubling of the odds per one SD decline in mtDNA-CN, compared with controls (OR = 1.76, 95% CI 1.19–2.60, *p* = 0.004). Additionally, we found a 32% increased odds for severe early AMD compared to AMD-free individuals with each decline by one SD in mtDNA-CN (OR = 1.32, 95% CI 1.07–1.62, *p* = 0.009). There was no significant association present for any of the remaining subtypes, especially not for macular neovascular AMD. When stratifying the analysis for men and women, we observed again the strongest association for geographic atrophy (OR = 1.88, 95% CI 1.07–3.32, *p* = 0.03 in women vs. OR = 1.54, 95% CI 0.93–2.54, *p* = 0.10 in men, Table 3). Also, the effect for severe early AMD was apparent in women and in men. On closer investigation of the different AMD subtypes, the effect directions were the same in both sexes in most subtypes. Notably, in the macular neovascular subtype, the effect directions differed between women (OR = 0.84, 95% CI 0.63–1.11, *p* = 0.22) and men (OR = 1.32, 95% CI 0.98–1.79, *p* = 0.15), and we identified a significant interaction with sex solely in this subtype (*p* = 0.006), which could explain the lack of significance in the combined analysis.

### 2.3. Mitochondrial DNA Haplogroup Analyses

As mitochondrial haplogroups were reported to play a role in AMD [17,18,19,20,21], we investigated whether mtDNA levels differed between haplogroups (distribution: 49.9% R0 cluster, 20.8% JT cluster, 21.0% UK cluster, 6.9% Other European and 1.3% Non-Europeans). Adjusting existing models for mitochondrial haplogroups had no influence on results (model 3, no vs. late AMD: OR = 1.23, 95% CI 1.03–1.46, *p* = 0.02). To further investigate potential mtDNA-CN variations associated with these haplogroups and AMD, we not only compared the mean mtDNA-CN between these five clusters, we additionally conducted subgroup analyses within each cluster, but only for subgroups with a sample size greater than 100 individuals (Table S2). However, neither the cluster-based analyses nor the subgroup-based analyses revealed any significant associations between mitochondrial haplogroups and mtDNA-CN in the different AMD groups (based on ANOVA and Kruskal–Wallis tests). Moreover, no significant difference between the five clusters of mitochondrial DNA and the case distribution of the three AMD groups (no vs. early vs. late AMD; χ^2^ *p*-value = 0.24, Table 1) or all AMD subtypes (χ^2^ *p*-value = 0.33) was found. Frequencies of all subhaplogroups are summarized in Table S3.

## 3. Discussion

In the study at hand, we demonstrated a significant association between mtDNA-CN in peripheral blood and AMD. Most interestingly, this association was most pronounced for geographic AMD (late AMD), with an almost doubling of the odds per standard deviation decrease in mtDNA-CN compared with AMD-free controls. It was less pronounced, but still significant, for severe early AMD compared with AMD-free controls, and it was not apparent for macular neovascular AMD or mild or moderate severe early AMD stages. Our results suggest that mtDNA-CN in blood can capture AMD risk in a similar fashion as mtDNA-CN in retinal tissue and might thus be a more easily accessible biomarker for AMD for epidemiological and clinical studies. Especially because blood sampling is less invasive and risky, and much more suitable for large-scale epidemiological studies in individuals with and without AMD, it represents a suitable alternative compared with obtaining retinal tissue via biopsy.

To our knowledge, this is the first large epidemiological study that investigated mtDNA-CN in peripheral blood for AMD. The previous literature is mainly based on experiments with tissue from donor eyes and cybrids or focuses on the identification of specific mtDNA variants instead of the mtDNA-CN [36,37,38]. One study used cybrids (eukaryotic cells where parts of the cellular components are exchanged from another cell) with mitochondria from either AMD patients or age-matched healthy subjects. They reported a reduction in mtDNA-CN of 31% in the cybrids with mitochondria from five AMD patients [37]. Another study demonstrated that treatment with humanin, a mitochondrial derived peptide, increased the mtDNA content in cultured retinal pigment epithelium cells and protected these cells from oxidative stress and senescence [36]. DNA was extracted from cybrids and retinal pigment epithelium cells, respectively, and mtDNA-CN was measured via qPCR in both of these studies [36,37]. Experiments with peripheral blood as testing material were rarely performed, and it has been shown that more mtDNA damage was present in the retina compared with blood samples [38]. While there is evidence for tissue dependency in mtDNA-CN levels [27], we show in the present study that mtDNA-CN in peripheral blood is associated with AMD. This suggests that mtDNA-CN in blood might be an accessible biomarker for mtDNA-CN in the retina. 

In our study, we demonstrate that mtDNA-CN is different between men and women, which is in accordance with literature [41,42,43]. There are various potential reasons for this phenomenon, including the influence of hormones such as estrogen, as well as environmental factors or specific genetic variants that have different effects on mtDNA-CN in men and women [41,42,43]. Adjustment by sex is thus important to avoid confounding. Statistically significant interaction was found only in macular neovascular AMD. Larger studies would be required to investigate the sex-specific interaction in this subtype and allow us to grasp the extent of the underlying association.

### 3.1. Biological Hypothesis

The retina is one of the most energy-demanding tissues in the human body. Therefore, the number of mitochondria in the retina is rather high compared with many other human tissues [44]. Photoreceptors have an especially high oxygen consumption, and, due to the loss of photoreceptors during the disease development and progression of AMD, oxidative damage is likely to appear. Consequently, the number of functional mitochondria is reduced, as mtDNA is less protected against DNA damage than nuclear DNA [24,25]. As a consequence of increased ROS levels and mitochondrial dysfunction, less energy can be produced, which influences not only the maintenance of membrane potential but can also initiate apoptosis via the release of the cytochrome complex. Moreover, it has been shown that mitochondrial dysfunction and retinal pathogenesis correlate, both on a genetic as well as a phenotypic level [15,18,20,24]. Different mitochondrial haplogroups that influence the risk for AMD and SNPs associated with mitochondrial genes such as NADH dehydrogenase or ubiquinone oxidoreductase have an effect on AMD [17,18,19,20,21,45,46]. Several mitochondrial variants, mainly in the D-loop of the mtDNA, have been found to be associated with AMD [38]. Mitochondrial diseases can present itself as an impairment in the retina. Therefore, it is discussed to use mtDNA-CN as an indicator for the severity of mitochondrial dysfunction and as clinical biomarker for the damage in the retinal tissue [47]. 

While mtDNA-CN in the retina has thus been documented to be a biomarker for oxidative stress damage related to AMD, our results suggest further that mtDNA-CN in blood might also be a marker. MtDNA-CN in blood is, in contrast to mtDNA-CN in the retina, easily accessible by peripheral blood draw. This enables applications in epidemiological studies and clinical trials. The fact that the association was only present for geographic atrophy and not for macular neovascularization, and thus for AMD-linked cell damage rather than neovascularization, is a further indication that lower mtDNA-CN captures the cell damage in retina. Further studies are warranted to evaluate the change in peripheral blood mtDNA-CN by geographic atrophy progression and the use of blood mtDNA-CN as a biomarker. 

### 3.2. Strengths and Limitations 

To our knowledge, this is the largest association study of peripheral blood mtDNA-CN and AMD [36,37,38]. One strength is the study design, as all participants in the population-based AugUR study were above 70 years old and from the same geographical area in Regensburg, Germany. All study participants, AMD patients and AMD-free controls were recruited and examined with the same standardized protocol. All mtDNA-CN measurements were performed in a blinded fashion, under the exact same conditions and within a short time frame in the laboratory at the Medical University of Innsbruck. Another strength of this study is that the AMD grading was performed by an experienced ophthalmologist based on color fundus photographs. 

Using the same DNA extraction method throughout an entire study is of importance, since we demonstrated recently that the DNA extraction method has a huge impact on the measurement of mtDNA-CN [48]. Therefore, limitations of this study include the two different salting-out methods applied for extraction of the DNA, leading to different effects on mtDNA and nuclear DNA during the centrifugation and incubation steps. Taking this issue into account, we analyzed the entire study with mixed-effect models to account for the differences. Additionally, over 96% of participants of this elderly study sample reported medication intake and the majority reported comorbidities. This makes the identification of selective predictors of interest more complicated. Furthermore, we want to emphasize the necessity of accounting for blood cell counts due to the varying levels of mtDNA in different types of blood cells. For instance, if a blood sample contains a higher proportion of leukocytes with elevated mtDNA-CN, it could lead to an artificial inflation of the overall measurement of mtDNA-CN. The same applies to high levels of thrombocytes, as those do not possess a nuclear genome. Therefore, we made model 3 our main model, which adjusts for age, sex, smoking, and leukocyte and thrombocyte counts. While we have made efforts to account for variations in blood cell counts, it is important to acknowledge that mtDNA-CN might be still influenced by the compositions of different cell types. This remains a significant challenge in accurately determining mtDNA-CN overall. Finally, although we cannot infer causation from our analyses, our models represent a novel contribution to the understanding of oxidative stress in the AMD pathogenesis, showing alterations of mitochondrial function present in peripheral blood that might be a marker for alterations in retina. Further studies would be interesting to help us understand whether alterations in mitochondrial function that are measurable in blood could be part of the cause rather than a consequence of AMD. 

## 4. Materials and Methods

### 4.1. Study Design, Data Collection and AMD Grading 

The University of Regensburg in Germany started the population-based AugUR study (Altersbezogene Untersuchungen zur Gesundheit der University of Regensburg) in 2013 with the aim of investigating age-related traits on a genetic and non-genetic level. Details on the study design have been published elsewhere [49]. Briefly, the study includes 2449 participants from the mobile elderly population of Regensburg. All participants were at least 70 years old at the time of enrollment. Information on sociodemographic data, lifestyle, metabolic parameters, medication intake, and general and ocular morbidities was collected via interviews and medical exams [49]. For ophthalmological assessment, non-stereo color fundus photography was conducted using the automatized Digital Retinography System (DRS) camera (CenterVue, Padova, Italy) after administering a mild mydriasis as described previously [4]. At least two images of each eye were acquired, and the central or central nasal fields of the retina were captured within a 45° view. An experienced ophthalmologist manually graded the color fundus images applying the Three Continent AMD Severity Scale (no AMD, mild/moderate/severe early AMD, late AMD) [39]. An experienced and trained ophthalmological consultant (C.B.) graded the color fundus images, and questionable findings were discussed with a second trained grader (ophthalmological consultant). Intergrader reliability was assessed via an independent grading by the second grader with a concordance of 95.3% and quadratic weighted kappa of 0.972. AMD status per person was based on the more diseased eye [4,49]. Here, we analyzed the AugUR study participant data with successfully measured mtDNA-CN and available AMD status from baseline in cross-sectional analyses. 

### 4.2. DNA Extraction

As described previously, DNA extraction methods influence the results of mtDNA-CN measurements [48]. For the first third of the samples, the extraction was performed with Puregene (Qiagen, Hilden, Germany) reagents. Due to high fluctuations in DNA concentrations and narrow yield in this elderly study sample, the extraction method was changed to a similar salting-out protocol [50] for the remaining samples. The differences between the two methods mainly involved extended incubation periods and a protein precipitation step carried out at 40 °C in the manual salting-out protocol to avoid SDS precipitation. Since mtDNA is generally less stable than nuclear DNA, longer incubation times and higher temperatures could potentially contribute to the degradation of mtDNA, resulting in lower levels of mtDNA-CN compared to those of the Puregene protocol. To investigate potential differences between the two DNA extraction methods on the mtDNA-CN measurements, DNA from 18 whole blood samples was extracted with both methods. A Bland–Altman plot (Figure S2) visualized a shifted mean value of the difference (−10.5) and a broad confidence interval. Correlation analyses revealed a correlation coefficient of 0.30 (*p* = 0.22). We therefore decided to treat the two subsets as independent study samples, and the DNA extraction method was considered as a random effect within the statistical analyses in multinomial mixed regression analyses. 

### 4.3. mtDNA Copy Number Measurements and Mitochondrial Haplogroup Determination

A previously described quantitative PCR (qPCR) approach with additional plasmid-normalization was used for mtDNA-CN measurements [48]. Briefly, by simultaneously using beta-2-microglobulin as the target for nuclear DNA and t-RNA^Leu^ as the target for mtDNA, the ratio between both targets can be measured. In contrast to other mtDNA-CN detection methods, this assay allows a reduction of inter-assay variability due to the use of a dual insert plasmid containing both targets. Samples were measured in triplicates and no modifications to the original protocol were made [30,48]. Three-fold standard curves verified efficiencies for both targets when measured in quadruplicates. A mean inter-assay variability of 5.3% was calculated based on two positive controls included in each of the 27 independent experiments. Four samples were excluded (0.17%), because the mtDNA-CN could not be evaluated properly and additionally, five outliers were excluded based on mtDNA-CN values beyond three standard deviations.

DNA samples were analyzed using the Illumina Global Screening Array v1/v3 for the AugUR Study. The quality of the genotype data was assessed using the standard parameters recommended by the GenCall software (GenomeStudio version 2.0) from Illumina (San Diego, CA, USA). Subsequently, the BIM, FAM and BED files were converted to VCF files with vcfCooker (version 1.1.1) https://genome.sph.umich.edu/wiki/VcfCooker. Genotypes that were missing (“./.”) were removed from the analysis. Subsequently, the VCF files were analyzed with HaploGrep2 (v2.4) [51] by specifying the chip parameter for limiting the input range to the present SNPs on the array. Using HaploGrep2, mitochondrial haplogroups were determined based on Phylotree 17 [52] and grouped in five consensus groups to allow analysis with sufficient power: (1) R0 including haplogroups R0, H, V and HV, (2) JT including macrohaplogroups J and T with all subhaplogroups, (3) UK including all U and sub-haplogroups including K, (4) other Europeans with haplogroup N1 (haplogroup I), N2 (haplogroup W) and X, and (5) Non-Europeans containing the remaining haplogroups (A, B, D, G, L, M, N8, R9). 

### 4.4. Statistical Analyses

In total, 2253 participants from the AugUR study with available mtDNA copy number measurements and AMD status were included in our final analyses. All analyses were performed using R 4.1.1 (Vienna, Austria, https://www.R-project.org) and a two-sided *p*-value < 0.05 was considered statistically significant. Baseline characteristics between participants with AMD and without AMD were compared using ANOVA or Kruskal–Wallis tests depending on the normal distribution of a variable. χ^2^ tests were used for categorical variables. Multinomial mixed models were applied using the AMD definition of the Three Continent Classification [39] as outcome (no, early, late AMD) to calculate odds ratios (OR) and to investigate the association of mtDNA-CN with AMD considering the two different DNA extraction methods (R package: mclogit, version 0.9.6). Different adjustment models were tested based on biological reasoning. Each model was adjusted for age and sex (if applicable), and further potential confounders were added, with the full model including the variables smoking, leukocyte and thrombocyte counts, cardiovascular disease (CVD), HDL cholesterol, hypertension, diabetes and HbA_1c_. Since age, sex, smoking and blood cell counts are the most important variables for adjusting mtDNA-CN, this set of adjustments was chosen as the main model (model 3 in all respective Tables). Additionally, sensitivity analyses based on the formula described by Hurtado-Roca and colleagues [40] were performed to further investigate the role of leukocyte and platelet counts on mtDNA-CN. Moreover, a spline for logistic regression with AMD/no AMD as outcomes was created to evaluate the shape of the relationship between mtDNA-CN and AMD risk. A priori power calculations based on the available sample size and with early or late AMD as the outcome revealed a power of 80% to detect odds ratios between 1.1 and 1.3 per standard variation alteration of mtDNA copy number. 

## 5. Conclusions

In conclusion, we found an association between lower mtDNA-CN in peripheral blood and late AMD (most prominent in geographic atrophy). This study provides evidence that mitochondrial dysfunction in AMD is not limited to the retina alone, but is also reflected in other body fluids. These findings offer new perspectives and contribute to our understanding of how oxidative stress and changes in mitochondrial function contribute to AMD. Still, further research involving functional studies on mitochondria is necessary following this epidemiological investigation. 

## Figures and Tables

**Table 1:** Baseline characteristics of participants without AMD, participants with early AMD (according to Three Continent Classification) and with late AMD.

	No AMD (n = 1630)	Early AMD (n = 453)	Late AMD (n = 170)
Sex, n (% male)	794 (48.7%)	198 (43.7%)	81 (47.6%)
Age (years)	77.6 ± 4.71 [74.0; 76.8; 80.6]	79.1 ± 5.1 [75.2; 78.5; 82.6]	81.9 ± 5.7 [77.7; 81.6; 86.4]
Smoking (current/ former/never), n (%)	90 (5.5%)/644 (39.6)/892 (54.9%)	20 (4.4%)/172 (38.1)/259 (57.4%)	16 (9.5%)/57 (33.9)/95 (56.5%)
Body Mass Index (kg/m^2^)	27.7 ± 4.5 [24.7; 27.2; 30.3]	27.6 ± 4.7 [24.6; 27.0; 29.8]	28.1 ± 4.4 [25.4; 27.7; 30.3]
Diabetes Mellitus, n (%)	333 (20.4%)	82 (18.2%)	43 (25.3%)
Total cholesterol (mg/dL)	218 ± 46 [185; 218; 248]	218 ± 48 [187; 217; 250]	215 ± 48 [184; 216; 243]
LDL cholesterol (mg/dL)	142 ± 35 [116; 141; 164]	140 ± 34 [116; 139; 163]	139 ± 35 [115; 140; 161]
HDL cholesterol (mg/dL)	60.7 ± 15.1 [49.6; 59.1; 69.8]	63.6 ± 16.6 [52.1; 61.7; 72.8]	59.4 ± 15.2 [47.6; 58.5; 68.9]
Triglycerides (mg/dL)	160 ± 84 [102; 140; 195]	149 ± 85 [92; 129; 183]	159 ± 93 [96; 141; 188]
Leukocytes (G/L)	6.5 ± 2.0 [5.3; 6.3; 7.4]	6.4 ± 1.6 [5.4; 6.2; 7.2]	6.8 ± 1.9 [5.6; 6.5; 7.5]
Thrombocytes (G/L)	241 ± 63 [199; 235; 275]	241 ± 64 [199; 237; 275]	240 ± 61 [197; 242; 277]
HbA_1c_ (mmol/mol)	39.9 ± 7.3 [35.5; 38.4; 42.1]	38.9 ± 6.7 [34.5; 37.7; 41.0]	41.2 ± 9.4 [35.5; 38.8; 45.3]
Hypertension, n (%) *	1174 (72.1%)	325 (71.9%)	131 (77.1%)
Cardiovascular disease, n (%) ^†^	343 (21.3%)	87 (19.4%)	53 (31.4%)
Mitochondrial haplogroups, n (%) ^‡^			
Haplogroup cluster R0	792 (48.6%)	247 (54.5%)	86 (50.6%)
Haplogroup cluster JT	354 (21.7%)	77 (17.0%)	38 (22.4%)
Haplogroup cluster UK	347 (21.3%)	90 (19.9%)	36 (21.2%)
Haplogroup cluster “Other Europeans”	112 (6.9%)	34 (7.5%)	10 (5.9%)
Haplogroup cluster “Non-Europeans”	25 (1.5%)	5 (1.1%)	0 (0%)
Mitochondrial DNA copy number	152.3 ± 43.3 [122.0; 145.0; 176.0]	147.8 ± 40.8 [120.4; 141.7; 169.6]	142.0 ± 42.6 [112.2; 135.8; 162.7]

For continuous variables, means ± SD [25th; 50th; 75th percentile] are shown. Abbreviations: AMD = age-related macular degeneration, LDL = low-density lipoprotein, HDL = high-density lipoprotein. * Hypertension was defined by systolic blood pressure ≥ 140 mmHg and/or diastolic blood pressure ≥ 90 mmHg and/or the use of antihypertensive drugs. ^†^ Cardiovascular disease is defined by infarctions, stroke and/or presence of stents or a bypass. ^‡^ Cluster “R0” = haplogroups R0, H, V and HV. Cluster “JT” = macrohaplogroups J and T with all subhaplogroups. Cluster “UK” = all U and sub-haplogroups including K. Cluster “Other Europeans” = N1, N2 and X. Cluster “Non-Europeans” contains the remaining haplogroups.

**Table 2:** Multinomial mixed regression analysis investigating the association of mitochondrial DNA copy number (decrease by one standard deviation, which equals an average reduction of 41.5 copies) and AMD using different adjustment models in all participants, and stratified for men and women.

mtDNA Copy Number	All (n = 2253)				Women (n = 1073)				Men (n = 1180)			
	OR (95% CI)	*p*	OR (95% CI)	*p*	OR (95% CI)	*p*	OR (95% CI)	*p*	OR (95% CI)	*p*	OR (95% CI)	*p*
	Early AMD vs. No AMD		Late AMD vs. No AMD		Early AMD vs. No AMD		Late AMD vs. No AMD		Early AMD vs. No AMD		Late AMD vs. No AMD	
**Model 1** adjusted for age	1.05 (0.95–1.16)	0.32	1.25 (1.06–1.47)	**0.008**	1.02 (0.90–1.17)	0.78	1.12 (0.90–1.41)	0.29	1.10 (0.95–1.28)	0.18	1.37 (1.07–1.76)	**0.01**
**Model 2** adjusted for age, sex, smoking	1.07 (0.97–1.19)	0.20	1.22 (1.03–1.43)	**0.02**	1.02 (0.89–1.17)	0.76	1.09 (0.87–1.36)	0.47	1.10 (0.95–1.28)	0.20	1.35 (1.06–1.72)	**0.02**
**Model 3** adjusted for age, sex, smoking, leukocyte, thrombocyte counts *	1.10 (0.98–1.23)	0.09	1.24 (1.04–1.47)	**0.02**	1.04 (0.89–1.21)	0.63	1.07 (0.84–1.36)	0.59	1.16 (0.99–1.36)	0.07	1.45 (1.11–1.90)	**0.006**
**Model 4** adjusted for age, sex, smoking, CVD, HDL-C, hypertension	1.10 (0.99–1.23)	0.07	1.20 (1.01–1.42)	**0.04**	1.02 (0.89–1.19)	0.74	1.03 (0.82–1.30)	0.79	1.19 (1.02–1.40)	**0.03**	1.41 (1.08–1.82)	**0.01**
**Model 5** adjusted for age, sex, smoking, diabetes, HbA_1c_	1.08 (0.97–1.21)	0.15	1.22 (1.02–1.44)	**0.03**	1.02 (0.89–1.18)	0.75	1.04 (0.83–1.32)	0.71	1.14 (0.98–1.32)	0.09	1.41 (1.08–1.84)	**0.01**
**Model 6** fully adjusted ^†^	1.11 (1.00–1.25)	0.06	1.23 (1.03–1.46)	**0.02**	1.05 (0.90–1.22)	0.56	1.05 (0.83–1.34)	0.68	1.18 (0.01–1.39)	**0.04**	1.45 (1.11–1.91)	**0.007**

* model 3 = main model. 111 individuals excluded due to missing blood cell counts. ^†^ fully adjusted: adjusted for age, sex, smoking, CVD, HDL-C, hypertension, diabetes, HbA_1c_, and leukocyte and thrombocyte counts. Abbreviations: OR = Odds Ratio, CI = Confidence Interval, *p* = *p*-value, AMD = age-related macular degeneration, CVD = cardiovascular disease, HDL-C = HDL cholesterol.

**Table 3:** Multinomial mixed regression analysis investigating the association of mtDNA copy number (decrease by one standard deviation) for each AMD subtype compared to AMD-free controls and stratified for men and women (adjusted for age, sex, smoking, and leukocyte and thrombocyte counts).

mtDNA Copy Number	n	All		Women		Men	
		OR (95% CI)	*p*-Value	OR (95% CI)	*p*-Value	OR (95% CI)	*p*-Value
		AMD vs. No AMD		AMD vs. No AMD		AMD vs. No AMD	
Mild early (early AMD)	203	1.02 (0.88–1.18)	0.83	0.96 (0.78–1.18)	0.68	1.07 (0.86–1.32)	0.54
Moderate early (early AMD)	126	1.10 (0.91–1.32)	0.34	1.06 (0.84–1.34)	0.63	1.13 (0.83–1.52)	0.44
Severe early (early AMD)	124	1.32 (1.07–1.62)	**0.009**	1.19 (0.90–1.58)	0.21	1.25 (0.95–1.65)	0.11
Geographic atrophy (late AMD)	37	1.76 (1.19–2.60)	**0.004**	1.88 (1.07–3.32)	**0.03**	1.54 (0.93–2.54)	0.10
Macular neovascular AMD (late AMD)	97	1.08 (0.87–1.33)	0.49	0.84 (0.63–1.11)	0.22	1.32 (0.98–1.79)	0.07
Combination of Geographic atrophy and Macular neovascularization (late AMD)	36	1.34 (0.95–1.91)	0.10	1.45 (0.85–2.47)	0.17	1.13 (0.73–1.75)	0.57

Abbreviations: OR = Odds Ratio, CI = Confidence Interval, AMD = age-related macular degeneration.

## Data Availability

The data presented in the study at hand are available from the corresponding author on reasonable request and after approval of the involved study.

## Supplementary Materials

The following supporting information can be downloaded at: https://www.mdpi.com/article/10.3390/ijms242216406/s1.
