# Supplementary material for: Systemic Evidence for Mitochondrial Dysfunction in Age-Related Macular Degeneration as Revealed by mtDNA Copy Number Measurements in Peripheral Blood
Source: Int J Mol Sci. 2023 Nov 16;24(22):16406. doi: 10.3390/ijms242216406 (PMC10671207; doi:10.3390/ijms242216406)
Supplement: Supplementary file 1 [file ijms-24-16406-s001.zip › ijms-2688112-supplementary.pdf]

Supplemental Material for

Systemic evidence for mitochondrial dysfunction in age-related macular degeneration as revealed by mtDNA copy number measurements in peripheral blood

Table S1: Multinomial mixed regression analysis investigating the association of mtDNA copy number (decrease by one standard deviation) for each AMD subtype.

**mtDNA copy number**	**Mild early (early AMD)** (n=203)		**Moderate early (early AMD)** (n=126)		**Severe early (early AMD)** (n=124)		**Geographic atrophy (late AMD)** (n=37)		**MNV (late AMD)** (n=97)		**Geographic atrophy + MNV (late AMD)** (n=36)	
	**OR (95% CI)**	**P**	**OR (95% CI)**	**P**	**OR (95% CI)**	**P**	**OR (95% CI)**	**P**	**OR (95% CI)**	**P**	**OR (95% CI)**	**P**
**Model 1** adjusted for age, sex	1.02 (0.89-1.16)	0.81	1.00 (0.84-1.18)	0.96	1.25 (1.03-1.51)	**0.02**	1.92 (1.37-2.71)	**<0.001**	1.05 (0.87-1.28)	0.59	1.24 (0.90-1.71)	0.19
**Model 2** adjusted for age, sex, smoking	1.02 (0.89-1.16)	0.82	1.02 (0.87-1.22)	0.75	1.25 (1.04-1.52)	**0.02**	1.98 (1.39-2.81)	**<0.001**	1.04 (0.86-1.27)	0.68	1.26 (0.91-1.74)	0.17
**Model 3** * adjusted for age, sex, smoking, leukocyte, thrombocyte counts	1.02 (0.88-1.18)	0.83	1.10 (0.91-1.32)	0.34	1.32 (1.07-1.62)	**0.009**	1.76 (1.19-2.60)	**0.004**	1.08 (0.87-1.33)	0.49	1.34 (0.95-1.91)	0.10
**Model 4** adjusted for age, sex, smoking, CVD, HDL-C and hypertension	1.04 (0.90-1.20)	0.62	1.05 (0.88-1.26)	0.58	1.33 (1.09-1.63)	**0.006**	1.89 (1.28-2.79)	**0.001**	1.04 (0.85-1.28)	0.68	1.27 (0.90-1.79)	0.18
**Model 5** adjusted for age, sex, smoking, diabetes and HbA_{1c}	1.01 (0.88-1.17)	0.84	1.05 (0.88-1.25)	0.62	1.31 (1.07-1.60)	**0.008**	1.80 (1.22-2.64)	**0.003**	1.05 (0.86-1.29)	0.62	1.31 (0.93-1.84)	0.12
**Model 6** fully adjusted †	1.04 (0.89-1.20)	0.64	1.10 (0.91-1.33)	0.31	1.35 (1.09-1.66)	**0.005**	1.81 (1.21-2.70)	**0.004**	1.06 (0.86-1.31)	0.57	1.31 (0.92-1.88)	0.14

* model 3 = main model; 111 individuals excluded due to missing blood cell counts.

† fully adjusted: adjusted for age, sex, smoking, CVD, HDL-Cholesterol (HDL-C), hypertension, diabetes, HbA_{1c}, leukocyte and thrombocyte counts

Abbreviations: OR = Odds Ratio, CI = Confidence Interval, P = p-value, AMD = age related macular degeneration, MNV = macular neovascular AMD, CVD = cardiovascular disease, HDL-C = HDL-Cholesterol

Table S2: Mean mtDNA-CN for AMD groups and mitochondrial haplogroups (haplogroup clusters* and (macro)haplogroups).

	**Mean mtDNA-CN**					
	**No AMD**		**Early AMD**		**Late AMD**	
**Cluster R0 (n=1125)**	150.5 ± 43.1 [119.7; 143.3; 177.0]		144.7 ± 38.9 [118.7; 140.5; 166.6]		138.0 ± 41.4 [107.2; 136.2; 162.1]	
Haplogroup H	150.6 ± 40.9 [119.7; 143.3; 177.4]	n=707	144.3 ± 36.9 [120.3; 141.1; 166.6]	n=220	138.9 ± 42.5 [107.2; 134.8; 164.4]	n=74
Haplogroups HV and V	145.3 ± 39.5 [118.4; 140.6; 171.3]	n=68	141.8 ± 50.8 [112.5; 126.4; 165.5]	n=23	132.4 ± 35.9 [105.2; 148.2; 159.3]	n=11
**Cluster JT (n=469)**	156.3 ± 42.6 [125.1; 148.5; 176.8]		153.6 ± 41.7 [123.5; 147.0; 183.6]		144.5 ± 42.5 [118.8; 135.3; 162.7]	
Haplogroup J	156.9 ± 40.7 [125.2; 153.5; 175.6]	n=151	155.8 ± 48.1 [122.6; 149.2; 184.1]	n=41	141.7 ± 30.0 [120.1; 135.0; 154.0]	n=13
Haplogroup T	155.8 ± 44.1 [125.4; 145.6; 178.6]	n=203	151.2 ± 33.3 [129.5; 144.3; 165.5]	n=36	145.9 ± 48.3 [111.4; 135.7; 168.6]	n=25
**Cluster UK (n=473)**	154.1 ± 43.8 [124.2; 148.5; 175.9]		153.6 ± 45.8 [121.3; 142.4; 182.3]		139.6 ± 41.8 [113.4; 126.3; 156.1]	
Haplogroup U	155.3 ± 44.3 [123.8; 149.6; 176.7]	n=257	153.0 ± 46.8 [119.9; 141.2; 173.4]	n=73	142.7 ± 45.0 [112.7; 128.6; 161.0]	n=30
Haplogroup K	150.6 ± 42.3 [127.3; 144.4; 175.7]	n=90	156.1 ± 42.8 [124.9; 147.9; 184.3]	n=17	124.0 ± 9.3 [116.5; 126.1; 129.4]	n=6
**Cluster "Other Europeans" (n=156)**	146.1 ± 42.9 [120.6; 139.4; 165.8]		142.4 ± 38.5 [118.2; 136.5; 161.2]		175.8 ± 47.2 [132.4; 174.4; 210.4]	
**Cluster "Non-Europeans" (n=30)**	155.8 ± 47.5 [124.1; 139.8; 176.1]		143.1 ± 28.2 [128.3; 132.8; 158.1]		-	

* Group "R0" includes haplogroups R0, H, V and HV. Group "JT" includes macrohaplogroups J and T with all subhaplogroups. Group "UK" includes all U and sub-haplogroups including K. Group "Other Europeans" includes haplogroup N1, N2 and X. Group "Non-Europeans" contains the remaining haplogroups.

Abbreviations: AMD = age related macular degeneration, P = p-value.

Table S3: Frequencies of all subhaplogroups in the AugUR study.

Subhaplogroup	N (% of total)	Subhaplogroup	N (% of total)	Subhaplogroup	N (% of total)
**A**	3 (0.13%)	**H50**	2 (0.09%)	**M**	2 (0.09%)
**C1**	1 (0.04%)	**H51**	4 (0.18%)	**N**	35 (1.55%)
**C4**	1 (0.04%)	**H6**	38 (1.69%)	**N1**	13 (0.58%)
**C5**	1 (0.04%)	**H61**	2 (0.09%)	**R**	2 (0.09%)
**D2**	3 (0.13%)	**H65**	5 (0.22%)	**R0**	21 (0.93%)
**D4**	6 (0.27%)	**H7**	12 (0.53%)	**R1**	2 (0.09%)
**F1**	1 (0.04%)	**H73**	3 (0.13%)	**T**	2 (0.09%)
**G2**	2 (0.09%)	**H81**	1 (0.04%)	**T1**	20 (0.89%)
**H**	358 (15.88%)	**H85**	1 (0.04%)	**T2**	242 (10.74%)
**H1**	347 (15.39%)	**H9**	1 (0.04%)	**U**	142 (6.30%)
**H13**	29 (1.29%)	**H94**	1 (0.04%)	**U1**	3 (0.13%)
**H14**	3 (0.13%)	**HV**	6 (0.27%)	**U2**	22 (0.98%)
**H15**	2 (0.09%)	**HV0**	1 (0.04%)	**U3**	10 (0.44%)
**H2**	78 (3.46%)	**HV1**	1 (0.04%)	**U4**	53 (2.35%)
**H22**	1 (0.04%)	**HV4**	1 (0.04%)	**U5**	121 (5.37%)
**H26**	4 (0.18%)	**I**	8 (0.35%)	**U6**	1 (0.04%)
**H29**	4 (0.18%)	**I1**	16 (0.71%)	**U7**	1 (0.04%)
**H3**	14 (0.62%)	**I2**	8 (0.35%)	**U8**	7 (0.31%)
**H32**	2 (0.09%)	**I3**	1 (0.04%)	**V**	76 (3.37%)
**H35**	1 (0.04%)	**I4**	10 (0.44%)	**V1**	5 (0.22%)
**H39**	1 (0.04%)	**J1**	166 (7.36%)	**V2**	4 (0.18%)
**H4**	28 (1.24%)	**J2**	39 (1.73%)	**V6**	4 (0.18%)
**H41**	2 (0.09%)	**K1**	92 (4.08%)	**V7**	4 (0.18%)
**H42**	1 (0.04%)	**K2**	21 (0.93%)	**W**	10 (0.44%)
**H46**	2 (0.09%)	**L1**	1 (0.04%)	**W3**	11 (0.49%)
**H49**	1 (0.04%)	**L2**	2 (0.09%)	**W5**	8 (0.35%)
**H5**	53 (2.35%)	**L3**	5 (0.22%)	**X2**	36 (1.60%)

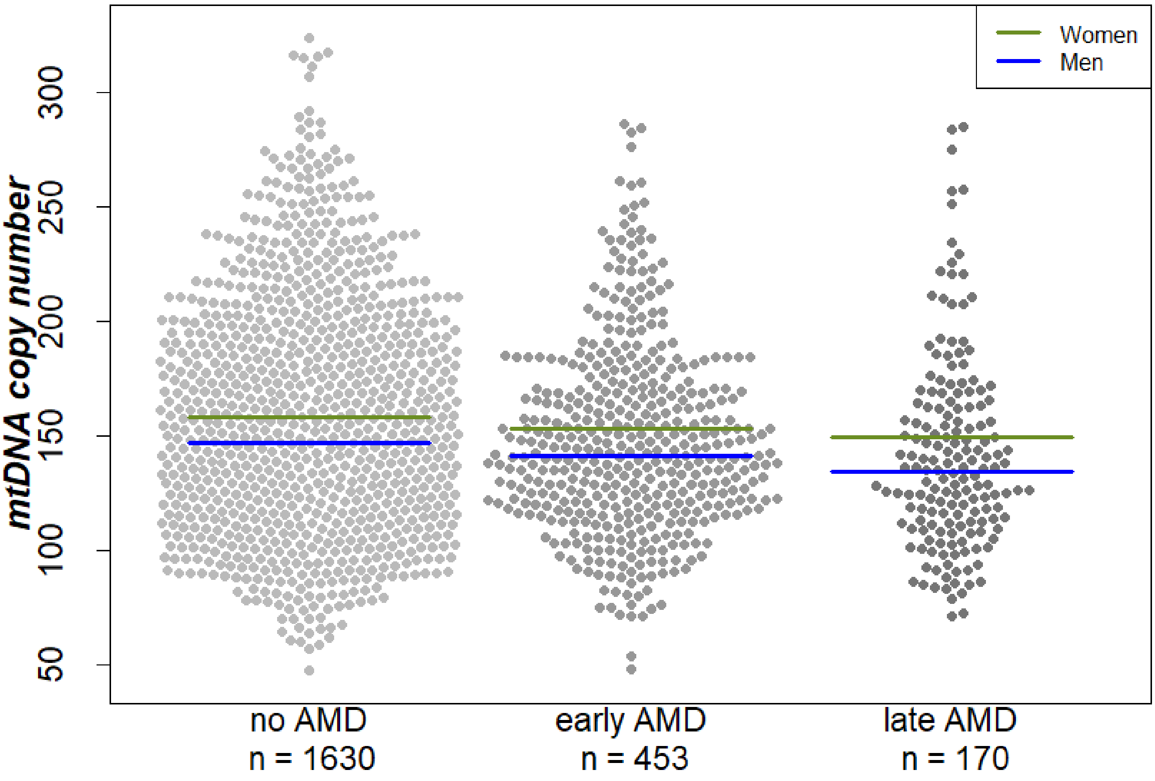

Figure S1: Distribution of mitochondrial DNA copy number in different AMD groups visualized in a beeswarm plot. Lines indicate the mean in the respective groups separated for men and women. For both, men and women, lowest mtDNA-CN was found in individuals with late AMD and the highest in AMD-free participants.

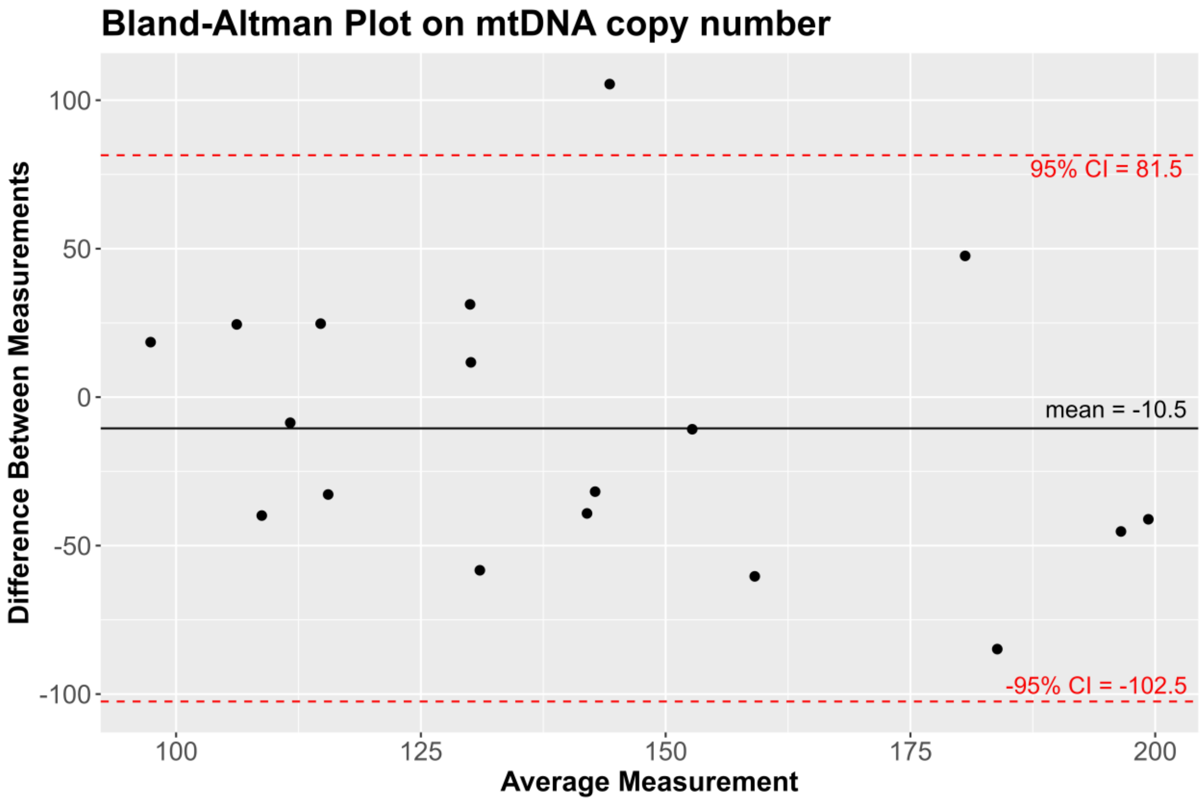

Figure S2: The agreement between both DNA extraction methods (Puregene vs. manual salting out protocol) is represented in this Bland-Altman plot based on the mtDNA copy number measurements of the same 18 samples. The black line represents the mean value of the difference between both measurements, the red dashed line the 95% confidence interval. On the x-axis, the mean value of both measurements is plotted. The y-axis demonstrates the difference between the two measurements. The mean mtDNA-CN of method 1 was 146.73 and 136.22 for the second extraction method.
